# Affective Impressions Recognition under Different Colored Lights Based on Physiological Signals and Subjective Evaluation Method

**DOI:** 10.3390/s23115322

**Published:** 2023-06-03

**Authors:** Xing Xie, Jun Cai, Hai Fang, Beibei Wang, Huan He, Yuanzhi Zhou, Yang Xiao, Toshimasa Yamanaka, Xinming Li

**Affiliations:** 1 School of Art and Design, Guangdong University of Technology, Guangzhou 510000, China; 2 Academy of Arts and Design, Tsinghua University, Beijing 100086, China; 3 Guangdong Provincial Key Laboratory of Nanophotonic Functional Materials and Devices, School of Information and Optoelectronic Science and Engineering, South China Normal University, Guangzhou 510006, China; 4 School of Physics and Telecommunication Engineering, South China Normal University, Guangzhou 510006, China; 5 Faculty of Art and Design, Tsukuba University, Tsukuba 305-8577, Japan

**Keywords:** colored light, mood, impression, GSR, ECG

## Abstract

The design of the light environment plays a critical role in the interaction between people and visual objects in space. Adjusting the space’s light environment to regulate emotional experience is more practical for the observers under lighting conditions. Although lighting plays a vital role in spatial design, the effects of colored lights on individuals’ emotional experiences are still unclear. This study combined physiological signal (galvanic skin response (GSR) and electrocardiography (ECG)) measurements and subjective assessments to detect the changes in the mood states of observers under four sets of lighting conditions (green, blue, red, and yellow). At the same time, two sets of abstract and realistic images were designed to discuss the relationship between light and visual objects and their influence on individuals’ impressions. The results showed that different light colors significantly affected mood, with red light having the most substantial emotional arousal, then blue and green. In addition, GSR and ECG measurements were significantly correlated with impressions evaluation results of interest, comprehension, imagination, and feelings in subjective evaluation. Therefore, this study explores the feasibility of combining the measurement of GSR and ECG signals with subjective evaluations as an experimental method of light, mood, and impressions, which provided empirical evidence for regulating individuals’ emotional experiences.

## 1. Introduction

Our emotional experiences and mood can be easily affected by external factors, such as light, sound, temperature, smell, and the space environment in which it is located [1]. In particular, creating a suitable space environment can enable individuals’ feelings or behaviors to meet more suitable functional needs for life, leisure, or work. Therefore, research on the influence of space design on human emotions and behaviors has attracted widespread attention. Among various external factors, lighting is an important and influential way to adjust the atmosphere of space to affect individuals’ moods. For example, natural light is often created to enhance the atmosphere for readers to concentrate on reading in libraries. The intensity of the light also affects individuals’ cognitive performance and is regulated by the type and difficulty of cognition [2]. The living room needs lighting to create a more comfortable atmosphere to alleviate the possible negative emotions of the occupants [3]. Generally speaking, illuminance and Correlated Color Temperature (CCT) play different roles in regulating emotional perception in indoor lighting [4]. In addition, the wavelength and intensity of light can also affect individuals’ alertness [5]. The color of light can affect individuals’ emotional changes, especially in pleasure and arousal [6]. In addition, the lighting arrangement will also affect the perception of space [7].

The object content, form, and space environment will be essential in the lighting atmosphere design [8]. Therefore, it is vital to study the impact of the lighting environment on individuals’ affective impressions of the target object. For example, exhibition halls, museums, etc. need to create effective interactions between people and objects, and light needs to stimulate individuals’ emotions and resonate with exhibits. A balance of warm and cool lighting choices is used for paintings [9]. The gallery’s lighting also affects the audience’s emotions and impressions of the artwork. Although the appreciation of works of art is a complex experience process, the CCT of lighting will significantly affect the affective impressions of people and works [10,11,12]. In actual museums, the study has found that high illuminance and high CCT have a higher psychological evaluation [13]. Although lighting plays an essential role in the space environment, evaluating the lighting for various objects and the resulting impact on individuals’ moods is still challenging to understand.

It is of great value to effectively evaluate the interaction effect of lighting on people and objects and to explore an individual’s emotional changes. For the observers, adjusting the light environment of the space to achieve the purpose of regulating emotions is more practical [14]. Subjective evaluation is often used to reflect the emotional impact of indoor lighting on individuals [15]. However, this method has certain limitations. For example, subjective evaluation is often the description of the participants after the experiment, which cannot realize the real-time evaluation of emotional changes. Thus, this post-event evaluation method is indirect. Therefore, real-time monitoring of individuals’ emotional changes under the light environment of space has become one of the crucial difficulties. More reliable and repeatable methods for the assessment of lighting effects are needed. Monitoring and evaluating emotions through physiological signals is an effective method, especially in some specific applications, such as autonomous driving, immersive experience, human–computer interaction, etc. [16,17]. This method is real-time when evaluating emotional changes because it can monitor the physiological information of the participants in the real interactive environment to assess the emotional states of the participants through physiological signals. For example, the electroencephalogram (EEG) signal is collected to form a dataset when a person watches a video to reflect the different emotional states of the participants [18]. Psychological information such as fingertip temperature and myoelectricity can also be applied to real-time dynamic objective emotional assessment [19].

Other synchronous physiological records obtained by sensors are intuitive and time-sensitive for emotional monitoring [20]. In recent years, GSR and ECG have been widely used in the emotional assessment of participants, and their effectiveness has been proven [21,22]. When a person is nervous, it often affects metabolism, and changes in sweat on the skin’s surface will affect the skin’s electrical signals. When a person has an enormous emotional change, the ECG signal will also experience related disturbances. The development of this technology also depends on the development of hardware equipment and the improvement of computing power [23]. Of course, this method also has specific difficulties, such as the physiological changes of the human reflected by the electrical signal itself. Still, the electrical signal changes induced by external stimuli cannot effectively distinguish the specific influencing factors brought about by external stimuli. Therefore, combining different methods is vital to comprehensively evaluate the influence of the light environment on individuals’ emotions by combining different methods.

Although the effects of lighting on an individual’s mood states have been extensively studied, the research on the effects of colored lights on mood is still insufficient, especially the effect of the interaction of colored lights with visual objects in the space on an individual’s emotions and cognition. This study proposes to combine subjective evaluation and physiological electrical signal (GSR and ECG) measurements to explore individuals’ mood evaluation of colored lights, and impressions toward images. The real-time monitoring of physiological signals could avoid the monitoring hysteresis and quantification errors in questionnaire surveys. It can also effectively evaluate the specific emotional impact of physiological signals. The research has the following hypothesis: Exposure to colored lights may affect an individual’s mood states, especially the type of visual objects in a colored light environment can affect an individual’s affective impressions.

## 2. Materials and Methods

### 2.1. Laboratory Setup

**Figure 1:**
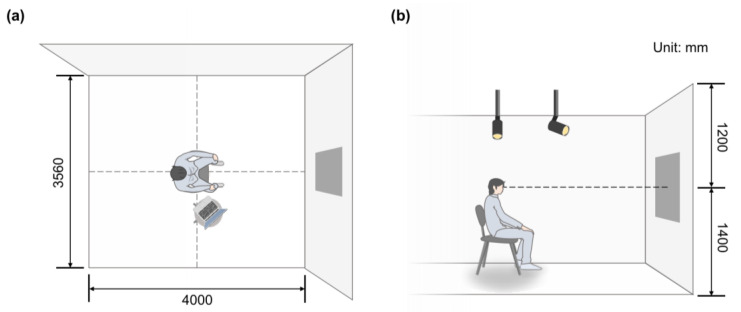
Schematic representation of laboratory simulation space. (**a**) Plan view; (**b**) elevation view.

To strictly control the effect of the experimental light on the subjects, the experimental darkroom with the size of 4 m × 3.56 m × 2.6 m was selected as the simulation space (see Figure 1). The visual center of the image was 1.4 m away from the floor. Without the influence of natural light, the relative humidity was maintained at 30% and the temperature was constant at 24 °C during the experiment. One lighting position was above the picture on a white wall, and the other was at the center of the ceiling to render the entire space. Related lighting parameters refer to our previous research [24].

### 2.2. Stimulus

To avoid possible interactive effects of the colors of images and those of lights on participants’ perception, monochrome images were used as stimuli. Moreover, the matte paper was used to prevent light reflection from affecting participants’ visual perception. These images were hung on the wall of the experimental darkroom after being printed on A3-sized papers.

**Figure 2:**
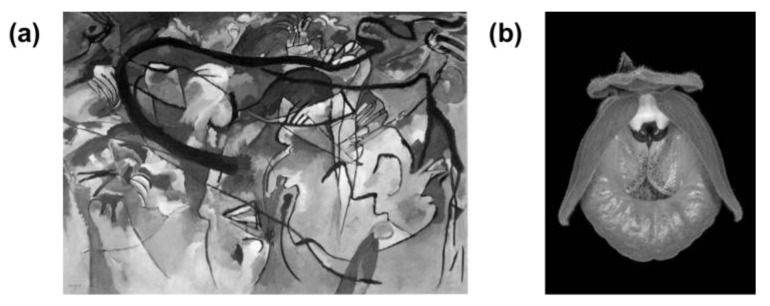
Abstract and realistic stimulus. (**a**) Kandinsky’s painting; (**b**) flower photography.

**Figure 3:**
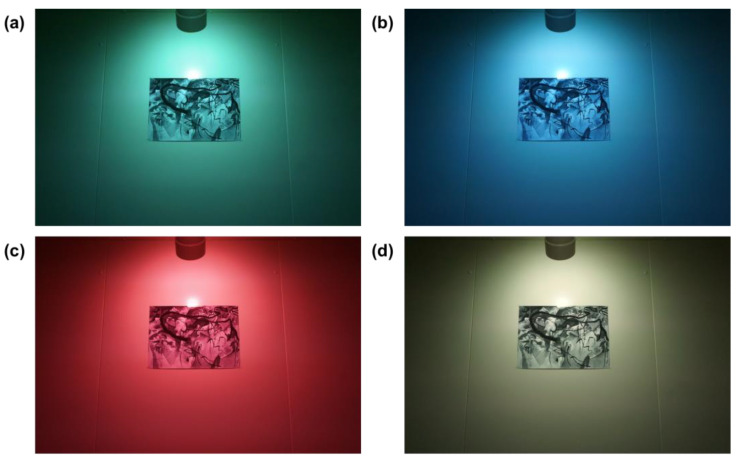
Stimuli under different lighting conditions. (**a**) Green light; (**b**) blue light; (**c**) red light; (**d**) yellow light.

In this experiment, considering the richness of realistic and abstract images, eight students from the university of China participated in the pre-experiment of material selection as volunteers through open recruitment (four male students and four female students, aged 20–35) and scored the matching degree of realistic and abstract images; six images were selected as experimental materials. Three represented abstract images, which are Kandinsky’s abstract painting, abstract animal (deer), and abstract view; the other three represented realistic images, which were realistic flowers, realistic animal (deer), and realistic view. Our previous research used four Kandinsky’s paintings and five flower photographs as abstract and realistic experimental materials, respectively [24]. In the present experiment, we chose two of these nine images as stimuli by material selection pre-experiment based on the previous study (see Figure 2). The display effects of the stimulus under different lighting conditions are shown in Figure 3.

### 2.3. Participants

Through snowball sampling, 16 Chinese observers aged 20–35 participated in the experiment as volunteers. Then, the final effective number was 14, including 8 males and 8 females. The observers provided written informed consent and received 25 RMB at the end of the study. The study was conducted under the Declaration of Helsinki, and the protocol was approved by the Ethics Committee of the first author’s institution.

### 2.4. Data Acquisition

**Figure 4:**
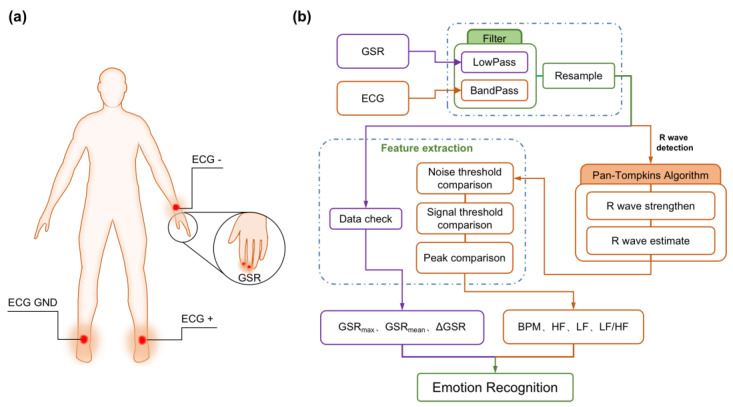
(**a**) Corresponding body parts for GSR and ECG signals collection; (**b**) schematic diagram of GSR and ECG signals processing.

To explore the individual’s response to the color lights, we collected GSR and ECG signals to represent the observers’ mood states when observers look at images under green, blue, red, and yellow light. As shown in Figure 4, both signals were recorded by the BIOPAC system (BIOPAC MP160) with the function of 16-channel physiological signal acquisition in a non-invasive manner. The GSR signal was collected using the EDA 100D intelligent amplification module. Two disposable electrode patches connecting the wires were attached to the distal phalanx of the index finger and middle finger, respectively. Observers maintain calm and should not shake as much as possible during the acquisitions. GSR signal is weak in the background of strong noise, which is interfered with by power frequency noise, electromagnetic interference, and motion artifacts. However, the effective frequency of the GSR signal is lower than most noise signals, so Low-Pass filtering can be used to filter out interference [25]. The amplitude of the GSR signal ranges from about 5 to 50 microsiemens (μS) and the frequency range is 0.02–0.2 Hz. The signal is filtered by FIR (Finite Impulse Response) low-pass filtering in the software Acqknowledge 5.0 supporting the acquisition device BIOPAC, and the Low-Pass cutoff frequency is 1 Hz (Blackman-92 dB). Then, the signal is resampled to 125 Hz. Finally, the maximum and average values of the signals of the corresponding time are extracted. The GSR signal is its transient change response as the stimulus response. Furthermore, the baseline of each person is quite different, so the difference between the maximum GSR (GSR-max) value during the picture stimulation process and the mean GSR (GSR-mean) in the calm state ΔGSR is taken as the index to measure the picture stimulation. The changing amplitude of the GSR signal is specifically realized as the absolute value of ΔGSR.

The intelligent ECG amplification module collected the ECG signal through a standard lead. Three disposable chip electrodes were used to connect the positive, negative, and ground wires, respectively (the left hand is negative, the left foot is positive, and the right foot is grounded). The raw ECG signal was analyzed by the software Acqknowledge 5.0. The process was divided into the filter [26], resample, R wave detection [27,28], and feature extraction of the preprocessed signals. We obtain features after peak, signal, and noise threshold comparisons. The time-domain features, such as heart rate (beat per minute, BPM), RR interval, and the frequency-domain feature, such as High-Frequency Power (HF), Low-Frequency Power (LF), and their ratio (Low-Frequency Power/High-Frequency Power, LF/HF) were extracted in each acquisition stage.

Moreover, the purpose of collecting self-report data was to learn more about the mood states and impressions of the observers. The design of the experimental questionnaire consisted of two parts. The first part was to obtain the individual’s mood response to colored lights through a Two-Dimensional Mood Scale (TDMS) (see Table 1). This method is to evaluate eight mood states before and after different light interventions, which are calm (M1), irritated (M2), lethargic (M3), energetic (M4), relaxed (M5), nervous (M6), listless (M7), lively (M8). Meanwhile, four levels of results were obtained through mathematical calculations, which were vitality (V), stability (S), pleasure (P), and arousal (A). The second part is an impressions evaluation scale, a five-point Likert scale (see Table 2). The observers evaluate the five questions by choosing “1: Not at all” to “5: Totally agree”, to obtain the individual’s subjective perception of the images under different lighting conditions.

### 2.5. Procedure

**Figure 5:**
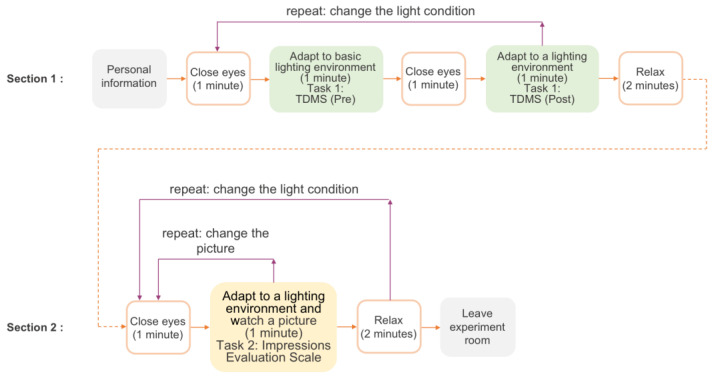
The procedures of the experiment.

As shown in Figure 5, the observers entered the waiting area, completed essential basic information and informed consent, learned the relevant experimental instructions, and started the experiment by wearing the physiological sensor investigation. The experiment was composed of two sections. In Section 1: (1) The observers completed the TDMS (pre) under the basic light (3500, CCT) to obtain the emotional data before being affected by the color light. (2) The observers closed their eyes for 1 min and then opened their eyes. After adapting to the lighting condition set by the testers for 1 min, they filled in the TDMS (post) to evaluate the emotions affected by the colored light. (3) Repeat steps (1) to (2), and the testers changed the lighting conditions until the observers finished watching tasks in all the lighting conditions, and then the observers closed their eyes to rest for 2 min. The sequence of lighting conditions was random for each observer. In Section 2: (4) The observers watched the images under the lighting conditions for 1 min and completed the impressions evaluation scale toward the picture. (5) The observers closed their eyes and rested for 1 min while the testers changed the pictures. (6) Repeat steps (4) to (5), and the tester changed the images until the observers watched all images toward one light condition, and then the observers closed their eyes and rested for 2 min. (7) Repeat steps (4) to (6), and the tester changed light conditions to ensure that each observer received four light conditions under six pictures. The order in which the pictures were displayed was random for each observer. All subjective questionnaire data were filled in on the E-prime program.

## 3. Results

Data were analyzed using SPSS 25.0 and jamovi. In terms of mood toward colored lights, a repeated measures ANOVA method was used to explore the changes in emotion before and after light stimulation. In terms of impressions toward images, a repeated measures ANOVA of 2 (image type: realistic and abstract) × 4 (lights: green, blue, red, and yellow) was performed.

### 3.1. GSR Data Analysis and Results

**Figure 6:**
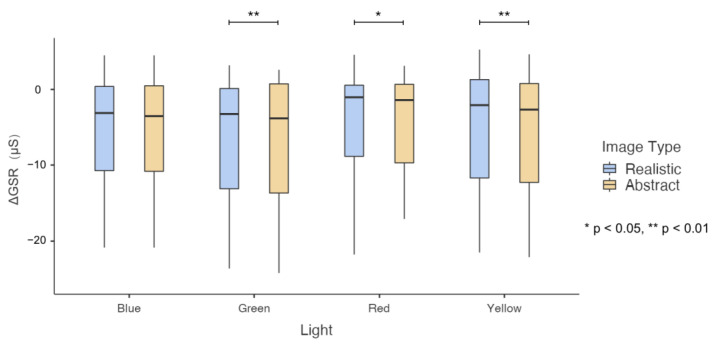
Results of participants’ ΔGSR signals toward images in colored light conditions.

A 2 (image type: realistic and abstract) × 4 (lights: green, blue, red, and yellow) repeated measures ANOVA was performed on GSR-max and ΔGSR data. The analysis only revealed significant differences in ΔGSR data. As shown in Figure 6, it was found that the main effect of image type was significant. The abstract images (M = −5.583, SD = 2.225) had significantly lower impression scores than the realistic images (M = −5.017, SD = 2.171), F(1, 13) = 3.661, *p* = 0.003, Partial Eta Squared = 0.512. The main effect of light was also significant, F(1, 13) = 3.661, *p* = 0.001, Partial Eta Squared = 0.555. There were significant differences among the four light conditions for both abstract pictures, F(3, 11) = 8.691, *p* = 0.003, Partial Eta Squared = 0.703 and realistic pictures, F(3, 11) = 6.619, *p* = 0.008, Partial Eta Squared = 0.891. The interaction effect between image types and light conditions was also significant, F(1, 13) = 3.661, *p* = 0.045, Partial Eta Squared = 0.274. Further simple effect analysis found abstract images (M = −5.424, SD = 2.174) had significantly lower ΔGSR than realistic images (M = −5.22, SD = 2.174) under blue light, F(1, 13) = 13.533, *p* = 0.003, Partial Eta Squared = 0.15; the ΔGSR of abstract images (M = −5.184, SD = 2.274) and realistic images (M = −3.919, SD = 2.074) was significantly lower under red light, F(1, 13) = 6.210, *p* = 0.027, Partial Eta Squared = 0.323. The ΔGSR of abstract images (M = −5.384, SD = 2.316) was significantly lower than that of realistic images (M = −4.902, SD = 2.293) under yellow light, F(1, 13) = 10.256, *p* = 0.007, Partial Eta Squared = 0.441. The ΔGSR of abstract images (M = −6.341, SD = 2.284) was not significantly different from that of realistic images (M = −6.027, SD = 2.222) under green light. Moreover, repeated measures ANOVA was used to verify the effect of light on GSR. The results showed that different light colors had no significant effect on the GSR-max and ΔGSR of the observers.

### 3.2. ECG Data Analysis and Results

To detect the changes in ECG signals before and after the light intervention, the values of HF, LF, and LF/HF were tested by two related samples of non-parametric tests to test the differences in the following four conditions: red light intervention; blue light intervention; green light intervention; yellow light intervention. The results showed no significant difference in the value of HF, LF, and LF/HF before and after the four light interventions. In addition, the difference before and after S, HF, and LF stimulation was calculated to detect whether the ECG signals were affected by the four kinds of light. A repeated-measures ANOVA showed that the four types of light did not significantly affect the changes in HF, LF, and LF/HF values. Moreover, a 2 (images: realistic and abstract) × 4 (lights: green, blue, red, and yellow) repeated measures ANOVA was performed on HF, LF, and LF/HF, and none of the results were significantly different.

### 3.3. Self-Reported Data Analysis and Results

#### 3.3.1. Colored Lights and Mood

Two related samples of non-parametric tests were performed to analyze the differences in the four colored light interventions. The results of the TDMS before and after the intervention are shown in Table 3. Under different colored light conditions, some items have significant effects in the emotional sense. Red light had significant differences in the mood of calm (*p* = 0.046), irritated (*p* = 0.002), energetic (*p* = 0.009), relaxed (*p* = 0.006), nervous (*p* = 0.003), lively (*p* = 0.004), vitality (*p* = 0.003), stability (*p* = 0.002), and pleasure (*p* = 0.002). Under the condition of blue light, there were significant differences in the mood of energetic (*p* = 0.043), relaxed (*p* = 0.011), nervous (*p* = 0.005), lively (*p* = 0.011), stability (*p* = 0.018), and pleasure (*p* = 0.020). Under the conditions of green light, there were significant differences in the mood of lethargic (*p* = 0.032), energetic (*p* = 0.008), relaxed (*p* = 0.004), nervous (*p* = 0.021), stability (*p* = 0.013), and pleasure (*p* = 0.035). Under the conditions of yellow light, there were significant differences in the mood of energetic (*p* = 0.018) and vitality (*p* = 0.042).

#### 3.3.2. Colored Light and Impressions

**Figure 7:**
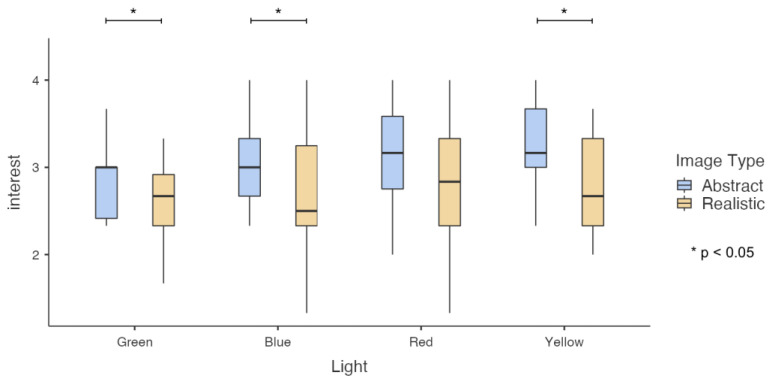
Results of participants’ impression of interest toward images in colored light conditions.

A 2 (image type: realistic and abstract) × 4 (lights: green, blue, red, and yellow) repeated-measures ANOVA was performed on five impressions, respectively. It was found that the main effect of image type on “preference” impression was significant. The impressive score of abstract image (M = 3.029, SD = 0.111) was significantly higher than that of realistic image (M = 2.756, SD = 0.16), F(1, 13) = 4.906, *p* = 0.045, partial Eta squared = 0.274. For the impression of “interest,” the main effect of image type was significant, and abstract images (M = 3.071, SD = 0.121) had significantly higher impression scores than realistic images (M = 2.684, SD = 0.153), F(1, 13) = 7.879, *p* = 0.015, Partial Eta Squared = 0.377. As shown in Figure 7, further simple effect analysis found that under green light, abstract images (M = 2.856, SD = 0.115) had significantly higher impression scores than realistic images (M = 2.571, SD = 0.118), F(1, 13) = 4.788, *p* = 0.048, Partial Eta Squared = 0.269. Under blue light, abstract images (M = 3.094, SD = 0.179) have a significantly higher impression score than realistic images (M = 2.666, SD = 0.21), F(1, 13) = 5.079, *p* = 0.042, Partial Eta Squared = 0.218. Under yellow light, abstract images (M = 3.214, SD = 0.159) had significantly higher impression scores than realistic images (M = 2.761, SD = 0.166), F(1, 13) = 7.415, *p* = 0.017, Partial Eta Squared = 0.363. Under red light, there was no significant difference in impression scores between abstract images (M = 3.119, SD = 0.166) and realistic images (M = 2.738, SD = 0.222).

**Figure 8:**
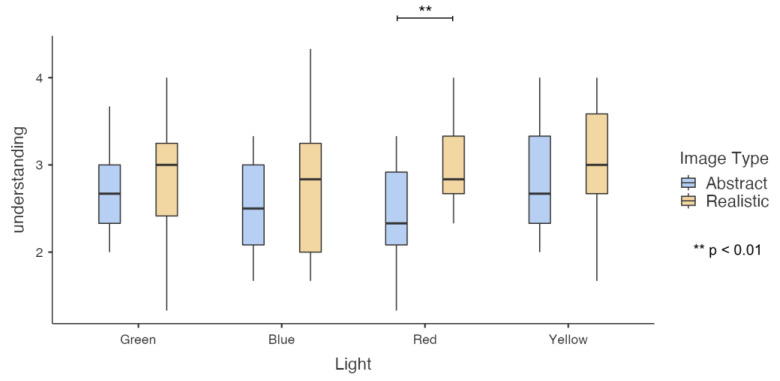
Results of participants’ impression of understanding toward images in colored light conditions.

For the impression of “understanding”, the main effect of image type was significant, and abstract images (M = 3.071, SD = 0.121) had significantly lower impression scores than realistic images (M = 2.613, SD = 0.129), F(1, 13) = 8.242, *p* = 0.013, Partial Eta Squared = 0.388. As shown in Figure 8, further simple effect analysis found that under red light, realistic images (M = 2.952, SD = 0.194) had significantly higher impression scores than abstract images (M = 2.428, SD = 0.15), F(1, 13) = 16.512, *p* = 0.001, Partial Eta Squared = 0.56. Under green, blue, and yellow light, there is no significant difference in impression scores between abstract images and realistic images.

### 3.4. Pearson Correlations between GSR Data, ECG Data, and Self-Reported Data

We further conducted correlation analysis of physiological signals using data of GSR-max, ΔGSR, HF, LF, and LF/HF, as well as ΔHF, ΔLF, and ΔLF/HF, which represented the HF, LF, and LF/HF difference before and after the stimulus. From the perspective of the effect of light on the mood, the mood of energetic correlated significantly with ΔHF (r = −0.328, *p* = 0.014) and ΔLF (r = −0.321, *p* = 0.016). For image-specific impression results, Table 4 shows the correlation between GSR and ECG signals: GSR-max was found to be significantly correlated with HF (r = −0.292, *p* <0.001) and LF (r = −0.276, *p* < 0.001), and ΔGSR correlated significantly with LF/HF (r = −0.194, *p* < 0.001). Table 5 shows the correlation between skin electricity, ECG, and impression. “Interest” impression was found to be significantly correlated with LF/HF (r = −0.145, *p* = 0.011). “Understanding” impression correlated significantly with ΔHF (r = −0.159, *p* = 0.005) and ΔLF (r = −0.142, *p* = 0.012). “Imagination” impression correlated significantly with GSR-max (r = 0.205, *p* < 0.001). Finally, the “feelings” impression correlated significantly with GSR-max (r = 0.173, *p* =0.002) and ΔGSR (r = −0.132, *p* = 0.02).

## 4. Discussion

Regarding physiological signal data results, the GSR signal detects that when abstract and realistic images are observed in a colored light environment, ΔGSR reflects the significant results of the interactive effects of colored light, image category, and the two. These results showed that compared with only being stimulated by light, the skin’s electric value is reduced, the sweat gland activity is reduced, and the sympathetic nerve activity is reduced. At the same time, the ΔGSR was lower when viewing abstract images than realistic images, indicating that the reduction of sweat gland activity and sympathetic nerve activity was reduced to a greater extent when viewing abstract images, and the ΔGSR difference between the two types of images is only shown under blue, red, and yellow light. There is no difference under the green light. The magnitude of the skin conductance response correlated with cognitive load measures in a previous study [29]. In addition, the three indicators extracted from the ECG signal failed to reflect statistically significant differences in emotions and impressions. This result may be due to the large differences in individuals’ physiological signal responses when stimulated [30]. To verify the corresponding relationship between the observers’ GSR and ECG signals, and the emotional perception of lights and images, it is necessary to compare the GSR and ECG data with the statistical results of the questionnaires.

According to the results of self-reported data, TDMS was once again proven to be an effective means for detecting transient changes in mood in subjective questionnaires. It is consistent with the previous research results that red light has the most significant effect on emotional arousal, followed by blue and green light. In contrast, yellow light has minimal effects [24]. Related results have also been reported in previous research, where musical performances under red light had higher perceived emotional expression than the same performance under blue light [31]. In this study, it is worth noting that red light, blue light, and green light can significantly decrease the mood of relaxation, stability, and pleasure, and substantially increase the mood of nervousness.

In contrast, these four mood states did not change significantly under the stimulation of yellow light. For the mood of energetic, the mood was significantly lowered under red, blue, and green light, while it was significantly increased under yellow light. This result may be because yellow light is close to the light in daily life, so it has a weaker effect on emotions than other colored lights. In addition, blue lights were rated more positively than red lights regarding overall mood ratings. Previous studies have also shown that blue is associated with more positive emotional experiences than red indoor environments [32,33], possibly because red light is perceived as more uncomfortable. Less spacious, related to warm colors making the space appear cramped [34]. Judging from the results of impressions when viewing images in a colored light environment, it is mainly the image itself that determines the impressions of preference, interest, and understanding and has nothing to do with lighting factors.

Regarding the overall score, abstract images are more difficult to understand than realistic images. Still, they are more attractive. It can be seen that the difference between realistic and abstract images is noticeable. Iigaya’s study (2021) [35] on natural human behavior introduced four image characteristics, including whether the image is abstract or realistic and exhibiting a preference for abstract art. Previous studies have explored that pleasure during viewing is first enhanced by arousal of factors such as image novelty, complexity, unfamiliarity, etc., but then decreases as arousal (especially complexity) becomes too strong [36,37]. Therefore, the relationship between the arousal intensity produced by image-related factors and pleasure arousal is worthy of further research and discussion. The research results also show that colored lights will regulate the individual’s impressions and perception of the image. An interesting result is that the “interest” impression of the two images differs significantly only under green, blue, and yellow light. As for the understanding of image content, the two groups of images were only significantly different under red light. This result may be because, in this experiment, red light is the most intense lighting condition for the emotional arousal of the observers. Related research reports also pointed out that higher-level factors of image appreciation are meaning, emotion, and interpretation of images. An individual’s preference for images is related to whether they can be easily perceived [37,38]. Colored light may affect the experience of observing and appreciating images by adjusting individual emotions and then affecting the perception of images.

From the perspective of the correlation between physiological signals and self-report, the change of HF and LF before and after light stimulation is significantly negatively correlated with the mood of energy. Both GSR and ECG signals may undoubtedly reflect an individual’s mood. Regarding the impressions of interest, understanding, imagination, and feelings toward images under light conditions, there is a negative correlation between GSR and ECG signals. It can be seen that physiological electrical signals may reflect specific changes in emotional cognition. Previous studies have confirmed that GSR has higher accuracy than ECG in identifying the three emotional states of happiness, sadness, and neutrality, especially the ability to distinguish between happy and sad states with at least 99% accuracy [21]. In comparison, this study showed no significant correlation between physiological signals and the subjective evaluation results of mood, possibly because the description words of mood states in the TDMS scale are more specific. In contrast, the generation process of physiological electrical signals is more complicated, possibly including a rich expression of multiple mood descriptors. This reflects the difficulty and importance of using physiological signals to detect specific mood states.

This study also proposed a novel experimental method in which the GSR and ECG measurements were jointly used to avoid the influence of limb movement on the signal. During the experiment, the spatial movement of the observers was restricted, which has a particular gap with the form of activities in the real space of the individual. As a result, higher requirements are put forward for applying physiological signal measurement methods. Firstly, the thin and light sensor design may reduce the foreign body sensation produced by the human skin on the measuring equipment, thereby reducing the physical and psychological stimulation. Secondly, the movable wireless sensor design can realize the free movement of the observers in space, which is closer to the form of activity in real space. The development of previous research on sensor design aimed at improving its convenience, wearing comfort, and accuracy for long-term comfortable wearing [39,40], may enable the physiological signal measurement method to be applied to richer scenes such as real exhibition halls, home environments, offices, and outdoors, and provides support for related experimental research on individual emotional experience in spatial light environments.

There are limitations to this study that should be taken into account when drawing conclusions. The first limitation of this study is that it was performed on a relatively limited number of college students. Due to the limited sample size and participants’ social and cultural backgrounds, our findings require caution in their interpretation. It would be insightful to see if the same outcomes could be found with people with other backgrounds. Furthermore, in terms of evaluating light and emotional impressions, this study has set up experimental conditions, which are representative to a certain extent. However, the influence of light on emotional impressions is complex. There may be other factors, such as the relative positions between the individuals and the visual objects, individual activity trajectory, etc. Finally, although this study used various methods to quantify and assess mood changes, mood itself is complex. Therefore, more methods are still needed to explore individuals’ emotional perceptions in more scenarios.

## 5. Conclusions

This study proposes a method for assessing the impact of light on an individual’s emotional impressions based on the combination of physiological electrical signals (GSR and ECG) and subjective evaluation. By adjusting the four color changes of the light, it is found that colored light has a regulating effect on emotional perception, with red lights eliciting the most substantial emotional arousal, then blue and green. In addition, when individuals observe images, the abstract and realistic categories of images significantly impact the preference, sense of interest, and understanding of individuals in observing visual objects and interacting with the stimulation of the colored light environment, reflected in the changes in individuals’ GSR and ECG signals.

Simultaneous comparison of physiological electrical signals with mood scales and impression evaluation scales helps to better understand the relationships between individuals, environments, and objects in the colored light environment. These findings may help designers better understand the application of colored lighting in space and provide more empirical evidence and theoretical implication for space design and experience design.

## Figures and Tables

**Table 1:** Two-dimensional mood scale (TDMS).

Eight Mood States of the Two-Dimensional Mood Scale (TDMS)			
M1	calm	M2	irritated
M3	lethargic	M4	energetic
M5	relaxed	M6	nervous
M7	listless	M8	lively
**Four Levels Based on TDMS Score** **s** **Calculation Results**			
V	vitality	V = M4 + M8 − M3 − M7	
S	stability	S = M1 + M5 − M2 − M6	
P	pleasure	P = V + S	
A	arousal	A = V − S	

**Table 2:** Impressions evaluation scale.

Five Questions of the Impressions Evaluation Scale		
Q1	preference	How much do you like this image?
Q2	interest	How interesting is this image?
Q3	understanding	How well do you understand this image?
Q4	imagination	Is this work arousing more imagination for you?
Q5	feelings	Does this image arouse more feelings for you?

**Table 3:** Changes in two-dimensional mood scale scores pre- and post-intervention.

		Red Light			Blue Light			Green Light			Yellow Light		
		Pre	Post	↑ or ↓	Pre	Post	↑ or ↓	Pre	Post	↑ or ↓	Pre	Post	↑ or ↓
M1	M	2.79	2.21	↓ * p = 0.046	3.93	3.5		3.62	2.93		3.07	3	
	SD	1.188	1.051		0.829	1.092		1.193	1.439		1.269	1.24	
M2	M	0.79	2.5	↑ ** p = 0.002	0.86	1.21		0.77	1.71		1.07	1.43	
	SD	0.975	1.345		1.231	1.188		1.166	1.49		1.492	1.399	
M3	M	0.93	1.71		0.71	0.86		1	1.79	↑ * p = 0.032	0.57	1.43	
	SD	0.917	1.267		1.326	0.663		1.155	1.578		0.938	1.399	
M4	M	2.64	1.57	↓ ** p = 0.009	2.57	1.5	↓ * p = 0.043	2.77	1.5	↓ ** p = 0.008	2.93	3.546	↑ * p = 0.018
	SD	1.151	0.938		1.222	1.092		1.013	1.286		1.269	1.437	
M5	M	3.21	1.5	↓ ** p = 0.006	3.57	2.07	↓ * p = 0.011	3.46	1.71	↓ ** p = 0.004	3.43	2.57	
	SD	0.893	1.16		1.016	1.141		0.967	1.267		1.089	1.399	
M6	M	0.71	2.5	↑ ** p = 0.003	0.64	2.21	↑ ** p = 0.005	0.77	2.29	↑ * p = 0.021	0.93	1.79	
	SD	0.726	1.225		1.016	1.141		0.725	1.49		0.997	1.528	
M8	M	2.86	1.43	↓ ** p = 0.004	2.07	0.93	↓ * p = 0.011	2	1.36		2.21	1.5	
	SD	1.406	0.852		1.016	1.141		1.155	1.008		1.626	1.286	
V	M	3.36	−0.43	↓ ** p = 0.003	3.07	0.36		2.21	−0.64		3.5	0.21	↓ * p = 0.042
	SD	3.296	3.131		3.689	2.24		3.332	4.325		3.546	3.423	
S	M	4.5	−1.29	↓ ** p = 0.002	6	2.5	↓ * p = 0.018	5.14	0.64	↓ * p = 0.013	4.5	2.36	
	SD	2.504	3.496		2.219	3.92		3.278	5.032		2.902	4.798	
*p*	M	7.86	−0.43	↓ ** p = 0.002	9.07	2.5	↓ * p = 0.020	7.36	0	↓ * p = 0.035	8	2.57	
	SD	4.622	5.413		5.298	4.637		5.611	8.357		5.477	6.618	

* *p* < 0.05, ** *p* < 0.01. ↑ The mean value before intervention is smaller than that after intervention, ↓ The mean value before intervention is greater than that after intervention.

**Table 4:** Correlation coefficient between GSR and ECG toward images in colored light conditions.

	GSR-Max	ΔGSR
HF	−0.292 ***	-
LF	−0.276 ***	-
LF/HF	-	−0.194 ***

*** *p* < 0.001.

**Table 5:** Correlation coefficient between GSR, ECG, and impressions toward images in colored light conditions.

	GSR-Max	ΔGSR	LF/HF	ΔHF	ΔLF
interest	-	-	−0.145 *	-	-
understanding	-	-	-	−0.159 **	−0.142 *
imagination	0.205 ***	-	-	-	-
feelings	0.173 **	−0.132 **	-	-	-

* *p* < 0.05, ** *p* < 0.01, *** *p* < 0.001.

## Data Availability

The data that support the findings of this study are available from the corresponding author.
